# Effects of particulate matter on inflammatory markers in the general adult population

**DOI:** 10.1186/1743-8977-9-24

**Published:** 2012-07-06

**Authors:** Dai-Hua Tsai, Nadia Amyai, Pedro Marques-Vidal, Jia-Lin Wang, Michael Riediker, Vincent Mooser, Fred Paccaud, Gerard Waeber, Peter Vollenweider, Murielle Bochud

**Affiliations:** 1 Institute of Social and Preventive Medicine (IUMSP), Lausanne University Hospital (CHUV), Biopôle 2, Route de la Corniche 10, CH-1010, Lausanne, Switzerland; 2 Department of Chemistry, National Central University, Jhong-Li, 32001, Taiwan; 3 Department of Medicine, Internal Medicine, CHUV, Lausanne, Switzerland; 4 Institute for Work and Health (IST), Lausanne, Switzerland; 5 Department of Genetics, GlaxoSmithKline, Philadelphia, PA, USA

**Keywords:** High-sensitive C-reactive protein (hs-CRP), Interleukin 1-beta (IL-1β), Interleukin 6 (IL-6), Tumor-necrosis-factor alpha (TNF-α), Air pollution

## Abstract

**Background:**

Particulate air pollution is associated with increased risk of cardiovascular disease and stroke. Although the precise mechanisms underlying this association are still unclear, the induction of systemic inflammation following particle inhalation represents a plausible mechanistic pathway.

**Methods:**

We used baseline data from the CoLaus Study including 6183 adult participants residing in Lausanne, Switzerland. We analyzed the association of short-term exposure to PM_10_ (on the day of examination visit) with continuous circulating serum levels of high-sensitive C-reactive protein (hs-CRP), interleukin 1-beta (IL-1β), interleukin 6 (IL-6), and tumor-necrosis-factor alpha (TNF-α) by robust linear regressions, controlling for potential confounding factors and assessing effect modification.

**Results:**

In adjusted analyses, for every 10 μg/m^3^ elevation in PM_10_, IL-1ß increased by 0.034 (95 % confidence interval, 0.007-0.060) pg/mL, IL-6 by 0.036 (0.015-0.057) pg/mL, and TNF-α by 0.024 (0.013-0.035) pg/mL, whereas no significant association was found with hs-CRP levels.

**Conclusions:**

Short-term exposure to PM_10_ was positively associated with higher levels of circulating IL-1ß, IL-6 and TNF-α in the adult general population. This positive association suggests a link between air pollution and cardiovascular risk, although further studies are needed to clarify the mechanistic pathway linking PM_10_ to cardiovascular risk.

## Background

Variations in short-term exposure to particulate matters (PM) have been repeatedly associated with daily all-cause mortality [1]. In the APHENA study, the combined effect of a 10-μg/m^3^ elevation in ambient particulate matters smaller than 10 μm (PM_10_) on all-cause mortality ranged from 0.2 % to 0.6 % [2]. Further, the exposure of older people to PM_10_ was more strongly associated with cardiovascular mortality (0.47 % to 1.30 %) than with non-cardiovascular mortality [2]. It was recently estimated that every 10 μg/m^3^ increase in the daily mean PM_10_ levels could be associated with a 1.6 % increased incidence of myocardial infarction [3]. Particle-induced inflammation has been postulated to be one of the important mechanisms for increased cardiovascular risk [1,4]. Experimental in-vitro, in-vivo and controlled human studies suggest that interleukin 6 (IL-6) and tumor-necrosis-factor alpha (TNF-α) could represent key mediators of the inflammatory response to PM exposure [1,5-7].

The associations of short-term exposure to ambient PM with circulating inflammatory markers have been inconsistent in studies including specific subgroups so far. Short-term exposure to PM has been associated with elevated C-reactive protein (CRP) in some [8-12], but not in all [13-15] studies. Short-term exposure to PM has also been associated with elevated IL-6 in some susceptible subjects [16,17] and with elevated TNF-α in healthy children [8].

The results of large-scale population-based studies have also been largely inconsistent. Short-term exposure to PM has been associated with elevated fibrinogen and CRP in some [18-20], but not in other [21-23] studies. Similarly, an association with elevated white blood cell counts was found in some [18], but not in other [22] studies. Hence, the epidemiological evidence linking short-term exposure to ambient PM and systemic inflammation in the general population is limited. So far, large-scale population-based studies have not explored important inflammatory markers such as IL-6 or TNF-α. We therefore analyzed the associations between short-term exposure to ambient PM_10_ and circulating levels of several inflammatory markers, namely high-sensitive CRP (hs-CRP), IL-1β, IL-6, and TNF-α in the population-based CoLaus study.

## Results

**Figure 1:**
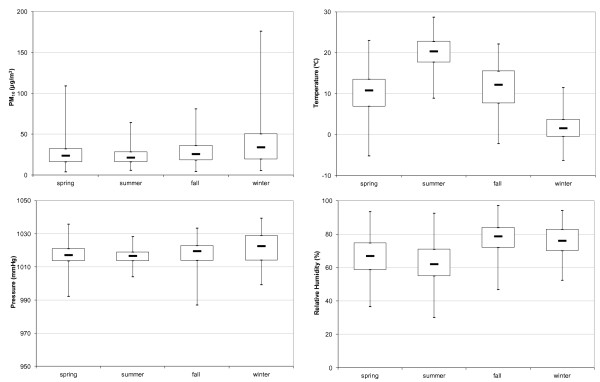
**Box plot of particular matter concentrations and selected meteorological data measured at the César Roux station during the period of the study from 2003 to 2006.** The median, 50^th^, and 75^th^ percentiles are given in the box plot. Whiskers show the maximum and minimum values.

The distributions of PM_10_, air temperature, relative humidity, and pressure by season are shown in Figure 1, with extreme values (minimum and maximum) and three quartile values (25, 50 and 75 %).

Participants’ characteristics are presented in Table 1. About 53 % were women and 57 % were older than 55 years. Women tended to have higher hs-CRP and IL-1β than men (p-value = 0.01 and <0.01, respectively), but significantly lower IL-6 and TNF-α (p-value <0.01). People aged 55 years and over had higher hs-CRP, IL-6 and TNF-α, compared with younger people. Similarly, overweight and obese participants, as well as those with hypertension, had higher hs-CRP, IL-6 and TNF-α levels, compared to their respective normal weight and normotensive controls. Smokers tended to have higher levels of all inflammatory markers. Diabetic participants and people who consume alcohol had higher values of hs-CRP, IL-6, and TNF-α. Sensitivity analyses led to similar results ( Additional file: Table S1).

**Table 1:** Levels of inflammatory markers overall and by selected subgroups

		**hs-CRP (μg/mL)**		**IL-1β (pg/mL)**		**IL-6 (pg/mL)**		**TNF-α (pg/mL)**	
	**Subjects [n]**	**N**	**Median (P25-P75)**	**N**	**Median (P25-P75)**	**N**	**Median (P25-P75)**	**N**	**Median (P25-P75)**
**All subjects**	6183	6171	1.30(0.60-2.70)	6083	0.39(0.10-1.74)	6077	1.32(0.58-3.21)	6077	2.87(1.79-4.50)
**Sex**									
Female	3251(52.6)	3247	1.30(0.60-2.90)	3200	0.45(0.10-1.93)	3197	1.21(0.53-2.93)	3196	2.73(1.71-4.38)
Male	2932(47.4)	2924	1.20(0.60-2.60)	2883	0.34(0.10-1.52)	2880	1.46(0.65-3.53)	2881	3.03(1.89-4.62)
p-value			0.01		<0.01		<0.01		<0.01
**Age (Years)**									
<55	3506(56.7)	3500	1.00(0.50-2.40)	3447	0.49(0.10-2.13)	3444	1.20(0.52-3.16)	3442	2.69(1.68-4.26)
> = 55	2677(43.3)	2671	1.60(0.80-3.20)	2636	0.29(0.10-1.32)	2633	1.46(0.68-3.27)	2635	3.13(1.99-4.82)
p-value			<0.01		<0.01		<0.01		<0.01
**BMI**									
<25	2970(48.0)	2963	0.80(0.40-1.70)	2923	0.46(0.10-1.99)	2921	1.15(0.49-3.10)	2920	2.68(1.68-4.20)
> = 25	3213(52.0)	3208	1.90(0.90-3.80)	3160	0.35(0.10-1.52)	3156	1.46(0.69-3.34)	3157	3.09(1.90-4.79)
p-value			<0.01		<0.01		<0.01		<0.01
**Smoking**									
No	4510(72.9)	4506	1.20(0.60-2.70)	4452	0.39(0.10-1.68)	4449	1.22(0.54-3.00)	4448	2.84(1.78-4.46)
Yes	1673(27.1)	1665	1.40(0.70-2.90)	1631	0.42(0.10-1.99)	1628	1.57(0.75-3.76)	1629	2.99(1.86-4.78)
p-value			<0.01		0.07		<0.01		<0.01
**Diabetes**									
No	5764(93.2)	5764	1.20(0.60-2.60)	5680	0.41(0.10-1.79)	5675	1.29(0.57-3.16)	5674	2.84(1.78-4.46)
Yes	407(6.8)	407	2.10(1.00-4.50)	402	0.15(0.10-0.97)	401	1.87(0.90-3.78)	402	3.52(2.20-5.35)
p-value			<0.01		<0.01		<0.01		<0.01
**Hypertension**									
No	3960(47.9)	3953	1.10(0.50-2.30)	3895	0.46(0.10-1.95)	3893	1.19(0.52-3.08)	3891	2.72(1.69-4.29)
Yes	2223(52.1)	2218	1.80(0.90-3.60)	2188	0.30(0.10-1.42)	2184	1.54(0.71-3.45)	2186	3.17(2.04-4.92)
p-value			<0.01		<0.01		<0.01		<0.01
**Alcohol**									
No	4613(74.6)	4606	1.20(0.60-2.70)	4541	0.42(0.10-1.83)	4536	1.27(0.55-3.10)	4536	2.84(1.78-4.50)
Yes	1567(25.4)	1567	1.40(0.70-2.70)	1540	0.34(0.10-1.58)	1539	1.48(0.66-3.54)	1539	2.96(1.84-4.60)
p-value			<0.01		<0.01		<0.01		0.11

Note: by Wilcoxon rank sum test.

**Figure 2:**
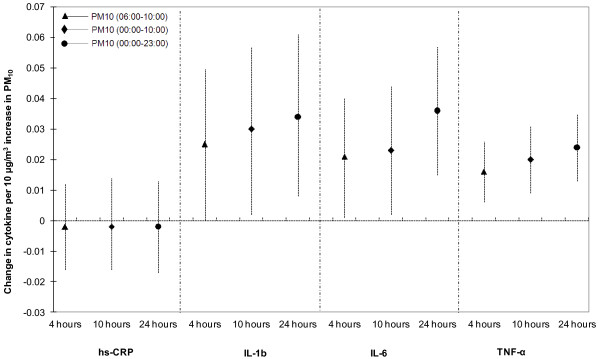
**Association between different time-averaged PM**_**10**_**concentrations and log-transformed inflammatory markers (adjusted effects).**

PM_10_ levels averaged over 24 hours were significantly and positively associated with continuous IL-1β, IL-6 and TNF-α levels in the whole study population both in unadjusted and adjusted analyses (Table 2). Except for IL-1β, sensitivity analyses led to similar results for IL-6 and TNF-α ( Additional file: Table S2). Secondary analyses using different time windows also led to similar conclusions (Figure 2). We found similar associations of PM_10_ with inflammatory markers with 1 to 6 days lag (Table 3). Sensitivity analyses led to similar results for IL-6 and TNF-α, but not for IL-1β ( Additional file: Table S3). Whereas the association of short term exposure to PM_10_ tended to decrease with increasing lag for TNF-α, no such tendency was observed for IL-1β or IL-6.

**Table 2:** **Change (and 95 % CI) in inflammatory markers associated with a 10 μg/m**^**3**^**change in ambient 24 h average PM**_**10**_**concentration**

	**Crude effects (95 % CI)**	**p-value**	**Adjusted effects (95 % CI)**^**a**^	**p-value**
**hs-CRP (μg/mL)**	0.004 (−0.015, 0.016)	0.961	−0.002 (−0.017, 0.013)	0.820
**IL-1β (pg/mL)**	0.040 (0.016, 0.065)	0.001	0.034 (0.007, 0.060)	0.014
**IL-6 (pg/mL)**	0.026 (0.006, 0.045)	0.010	0.036 (0.015, 0.057)	0.001
**TNF-α (pg/mL)**	0.015 (0.004, 0.025)	0.005	0.024 (0.013, 0.035)	<0.001

^**§**^ Covariates included in the model: sex, age, BMI, alcohol consumption, smoking status, education levels, zip code, statin, diabetes, hypertension, season, outdoor air temperature, and outdoor air pressure. Data are coefficients from robust regression models.

**Table 3:** **Association of short-term exposure to 24 h average PM10 with inflammatory markers on the day of examination, and with 1-day to 6-day lags from adjusted robust regression models**^**§**^

	**hs-CRP**	**p-value**	**IL-1β**	**p-value**	**IL-6**	**p-value**	**TNF-α**	**p-value**
**Lag 0 (Same day)**	−0.002	0.820	0.034	0.014	0.036	0.001	0.024	<0.001
	(−0.017, 0.013)		(0.007, 0.060)		(0.015, 0.057)		(0.013, 0.035)	
**Lag 1**	0.001	0.854	0.034	0.011	0.035	0.001	0.022	<0.001
	(−0.013, 0.016)		(0.008, 0.061)		(0.015, 0.056)		(0.011, 0.033)	
**Lag 2**	0.002	0.798	0.039	0.004	0.039	<0.001	0.023	<0.001
	(−0.013, 0.017)		(0.013, 0.066)		(0.019, 0.060)		(0.013, 0.034)	
**Lag 3**	−0.001	0.944	0.037	0.006	0.040	<0.001	0.023	<0.001
	(−0.015, 0.014)		(0.010, 0.063)		(0.020, 0.061)		(0.012, 0.034)	
**Lag 4**	0.002	0.784	0.038	0.004	0.041	<0.001	0.022	<0.001
	(−0.013, 0.017)		(0.012, 0.065)		(0.020, 0.061)		(0.011, 0.033)	
**Lag 5**	0.003	0.662	0.037	0.006	0.035	0.001	0.019	0.001
	(−0.011, 0.018)		(0.010, 0.063)		(0.015, 0.055)		(0.008, 0.030)	
**Lag 6**	0.002	0.753	0.030	0.025	0.036	0.001	0.019	0.001
	(−0.012, 0.017)		(0.004, 0.056)		(0.016, 0.056)		(0.008, 0.030)	

^**§**^Adjusted for: sex, age, BMI, alcohol consumption, smoking status, education levels, zip code, statin, diabetes, hypertension, season, outdoor air temperature, and outdoor air pressure.

The associations of PM_10_ with inflammatory markers in different subgroups are presented in Table 4. PM_10_ was significantly associated with IL-1β, IL-6 and TNF-α in men, but only with IL-6 and TNF-α in women. However, there was no significant statistical interaction between PM_10_ and sex. For IL-1β, IL-6 and TNF-α, the associations tended to be stronger in younger people. PM_10_ was significantly associated with IL-6 and TNF-α in the healthy group and also in the “non-healthy” group, although the statistical interaction between healthy status and PM_10_ was not significant. In addition, PM_10_ was significantly associated with IL-6 and TNF-α in the participants who were not on statin therapy. Sensitivity analyses led to similar results for IL-6 and TNF-α, but not for IL-1β ( Additional file: Table S4).

**Table 4:** **Associations of short-term exposure to 24 h average PM**_**10**_**with inflammatory markers, by selected strata**

	**hs-CRP**	**p-value**	**IL-1β**	**p-value**	**IL-6**	**p-value**	**TNF-α**	**p-value**
**Female**	−0.008	0.439	0.019	0.302	0.035	0.015	0.023	0.002
	(−0.028, 0.012)		(−0.017, 0.056)		(0.007, 0.063)		(0.008, 0.038)	
**Male**	0.009	0.416	0.050	0.012	0.041	0.009	0.025	0.002
	(−0.013, 0.031)		(0.011, 0.090)		(0.010, 0.071)		(0.009, 0.041)	
**Interaction p-value**^**§**^		0.560		0.376		0.869		0.620
**Age < 55**	−0.007	0.511	0.037	0.047	0.048	0.001	0.027	<0.001
	(−0.027, 0.013)		(0.0004, 0.073)		(0.020, 0.077)		(0.013, 0.042)	
**Age > =55**	0.007	0.527	0.030	0.141	0.022	0.144	0.020	0.021
	(−0.015, 0.029)		(−0.010, 0.069)		(−0.008, 0.052)		(0.003, 0.036)	
**Interaction p-value**^**§**^		0.404		0.703		0.105		0.619
**Healthy**^†^	0.025	0.151	0.038	0.250	0.062	0.022	0.035	0.009
	(−0.009, 0.058)		(−0.027, 0.103)		(0.009, 0.114)		(0.009, 0.062)	
**Non-healthy**^†^	−0.007	0.400	0.033	0.027	0.029	0.011	0.022	<0.001
	(−0.024, 0.009)		(0.004, 0.062)		(0.007, 0.052)		(0.010, 0.034)	
**Interaction p-value**^**§**^		0.620		0.708		0.862		0.769
**No statins**	−0.002	0.806	0.027	0.064	0.036	0.002	0.024	<0.001
	(−0.018, 0.014)		(−0.002, 0.056)		(0.013, 0.058)		(0.012, 0.036)	
**With statins**	0.006	0.773	0.073	0.057	0.038	0.165	0.020	0.203
	(−0.036, 0.049)		(−0.002, 0.015)		(−0.016, 0.093)		(−0.011, 0.051)	
**Interaction p-value**^**§**^		0.843		0.372		0.886		0.812

Data are coefficients (95 % CI) from robust regression models. Coefficients represent change in cytokine per 10 μg/m^3^ increase in PM_10_. ^**§**^ P-value for interaction between the strata variable and PM_10_ for their effects on the inflammatory marker.

^†^ Sample sizes for healthy and non-healthy are 993 and 5190, respectively.

## Discussion

In the population-based CoLaus study, short-term exposure to PM_10_ was associated with circulating levels of IL-1β, IL-6 and TNF-α, but not of hs-CRP. Our results are consistent with findings by Ruckerl et al., who showed no association of PM_10_ with hs-CRP, but a significant positive association of particle number concentration with IL-6 levels in 1003 myocardial infarction (MI) survivors [17]. In several small-sized studies, similar associations were found [24-26]. Furthermore, our results are in line with experimental data. Van Eeden et al. [24] showed that human alveolar macrophages produce TNF-α in a dose-dependent manner when exposed to atmospheric particles. Kido et al. [27] found the lung to be a major source of systemic circulating IL-6 levels in mice exposed to ambient PM. Our study is the first large scale population-based study to show significant positive associations of short-term exposure to PM_10_ with circulating IL-1β, IL-6 and TNF-α levels, which substantially increases the external validity of previous findings. The relevance of these results is emphasized by the fact that IL-6 plays a central role in the inflammatory response in the context of cardiovascular disease [5].

Systemic inflammation is known to be associated with cardiovascular morbidity and mortality [1]. Indeed, a number of epidemiological and experimental studies have shown that circulating markers of systemic inflammation and haemostasis are closely associated with the development of fatal and non-fatal MI [28-32]. Inflammation is likely to interfere directly with the blood coagulation pathways leading to hypercoagulation states [33], as well as to increase the probability for a plaque rupture leading to acute coronary events by accentuating atherosclerotic plaque vulnerability [28]. These findings, together with the observation that short-term exposure to higher PM_10_ levels are associated with the risk of myocardial infarction and acute ischemic stroke [34], suggest that air pollution-induced systemic inflammation may increase cardiovascular risk [3].

Many hypotheses have been proposed to explain the pathophysiological mechanism underlying the link between PM inhalation and systemic inflammation. Exposure of the pulmonary bronchial tree to PM may induce a local inflammatory reaction with the production of specific cytokines from neutrophils, macrophages and T cells [1]. In vitro studies have shown that human alveolar macrophages exposed to PM_10_ release numerous inflammatory cytokines, including IL-6 and TNF-α [6,7,24]. The diffusion of these cytokines in the systemic circulation induces a generalized reaction leading to the inflammation cascade [35]. Recently, a second hypothesis has been proposed, suggesting that inhaled PM and especially PM_2.5_ directly penetrate into the vascular tree where they interact with endothelial cells and the immune system further activating the inflammation cascade [36].

We found no association of short-term exposure to PM_10_ with circulating hs-CRP levels. This is in line with the results of previous population-based studies [20,21,23]. The absence of association with hs-CRP might be related to the fact that we only explored short-term exposure, as one study found a positive association of hs-CRP with long-term exposure to PM_10_[21]. Yet, Hertel at al found short-term exposure to particle number to be associated with hs-CRP in 4000 participants to the population-based Heinz Nixdorf Recall study [20]. CRP production is increased following the hepatic action of IL-6. CRP represents a later marker of inflammation than IL-6, for instance, with a half-life of around 15–19 hours for CRP [37]. This may explain the absence of association with short-term exposure to PM_10_ in our study.

This study has some limitations which should be acknowledged. This is a cross-sectional analysis using the absolute level of PM_10_ at a single point in time. Unfortunately, data for PM_2.5_ was not available in this study. Also, our results only pertain to short-term exposure to PM_10_. The effects of long-term exposure to PM_10_ levels could not be assessed because long-term individual-level exposure data are not available. Personal exposures can vary substantially from the levels measured at a central monitoring station. We did not capture differences in long-term exposure due to different exposures to local sources (roads, industrial sources). This is a problem common to large epidemiological studies that, unlike panel studies, cannot equip each subject with personal exposure monitoring devices. To account for variations attributable to spatial differences in participants’ place of residence, we included the zip code as a covariate in the models. PM_10_ is a complex mixture of chemical compounds the behavior of which strongly depends on the atmospheric conditions. Putard et al. reported that the most important compounds to PM_10_ mass in four Swiss locations (Bern, Zurich, Basel, and Chaumont) were black carbon, organic matter, mineral dust, ammonium, nitrate and sulphate [38]. The studied population is from a single geographical area, and the findings may not be generalisable to other regions or cities. In addition, results for IL-1β should be interpreted with caution because 38 % of IL-1β was below the detection limit of the assay. More sensitive assays are therefore needed to reduce the proportion of undetectable values for IL-1β and to provide better estimates of the association of PM_10_ with circulating IL-1β levels.

## Conclusions

In summary, we found significant independent positive associations of short-term exposure to PM_10_ with circulating levels of IL-6 and TNF-α in the adult population of Lausanne. Our findings strongly support the idea that short-term exposure to PM_10_ is sufficient to induce systemic inflammation on a broad scale in the general population. Even slight increases in the distribution of inflammatory cytokines may represent a substantial health burden at the population level, in particular by increasing cardiovascular morbidity and mortality, although the precise mechanistic pathway linking PM_10_ to cardiovascular risk has not yet been elucidated. From a public health perspective, the reported association of elevated inflammatory cytokines with short-term exposure to PM_10_ in a city with relatively clean air such as Lausanne supports the importance of limiting urban air pollution levels.

## Methods

### Study population

All study subjects were participants to the CoLaus study (www.colaus.ch), an ongoing population-based cohort study. The baseline examination was carried out from 2003 to 2006. Participants’ recruitment has been described in detail previously [39]. Briefly, the population registry of the city provided the complete list of the Lausanne inhabitants aged 35–75 years (n = 56,694). Subjects were selected using a simple, non-stratified random selection approach. Overall, 6184 participants were included. For the present analysis, 6183 participants had data on at least one of the four measured circulating inflammatory markers.

### Analytical method

Venous blood samples (50 mL) were drawn in the fasting state between 7 am and noon. Hs-CRP was assessed by immunoassay and latex HS (IMMULITE 1000–High, Diagnostic Products Corporation, LA, CA, USA) with maximum intra- and inter-batch coefficients of variation of 1.3 % and 4.6 %, respectively. Serum samples were kept at −80 °C before assessment of the other cytokines (IL-6, IL-1β and TNF-α) and sent in dry ice to the laboratory. Cytokine levels were measured using a multiplexed particle-based flow cytometric cytokine assay [40], a methodology used in other studies [41]. Milliplex kits were purchased from Millipore (Zug, Switzerland). The procedures closely followed the manufacturer’s instructions. The analysis was conducted using a conventional flow cytometer (FC500 MPL, BeckmanCoulter, Nyon, Switzerland). The lower detection limit for IL-1β, IL-6 and TNF-α was 0.2 pg/mL. There were 2388, 556 and 148 values below lower detection limits for IL-1β, IL-6 and TNF-α, respectively. A good agreement between signal and cytokine was found within the assay range (R^2^ ≥ 0.99). Intra- and inter-assay coefficients of variation were 15 % and 16.7 % for IL-1β, 16.9 % and 16.1 % for IL-6 and 12.5 % and 13.5 % for TNF-α, respectively. Repeated measurements conducted in 80 subjects randomly drawn from the initial sample showed very high reproducibility.

### Air pollution data

The monitoring station is located in Lausanne-César-Roux, at 530 meters above sea level, next to a slightly ascending inner city transit road (30,000 vehicles per day). The station is about 400 meters away from the University Hospital of Lausanne (CHUV) where the participants’ examination took part, always between 7 am and noon. On one side of the road is an open school house yard which favors good mixing of the air. In the close vicinity are exclusively apartment buildings and service companies.

The monitoring data was obtained from the website of Swiss National Air Pollution Monitoring Network (NABEL) [42]. We analyzed data on PM_10_ as well as outside air temperature, pressure and humidity. Hourly concentrations of PM_10_ were collected from 1 January 2003 to 31 December 2006, for a total of 1461 days. Data control and quality control were done regularly by the responsible agency. Hourly concentrations were then averaged into 24 hours means (0:00–23:00) as a point estimate of air pollutant levels in the study area. We matched air pollution and meteorological data to each participant’s examination day.

### Statistical analysis

We defined as extreme outlier cytokine values ten times higher than the value of the 99^th^ percentile. There was one extreme outlier for IL-1β, which was higher than 884 pg/mL (p99 = 88.4), 7 outliers for IL-6, which were higher than 1084 (p99 = 108.4) pg/mL, and 7 outliers for TNF-α higher than 500 pg/mL (p99 = 50.0). We excluded those values from the analyses. All values of IL-1β, IL-6, and TNF-α below the detection level (0.2 pg/mL), were substituted with a value (0.1) equivalent to half of the lower detection limit as recommended by Uh et al. [43]. We performed sensitivity analyses by setting values which were below the detection limits as missing. The results are shown in the Additional files.

Robust linear regression was used to evaluate the relationship between cytokine inflammatory and PM_10_. Robust regression is a family of regression methods for data in which non-robust least-squares methods may be biased by outliers or heteroscedasticity. The method we chose for estimation was Huber M estimation. We adjusted all analyses for age, sex, body mass index (BMI, calculated as weight in kilograms/height in meters squared), smoking status, alcohol consumption, diabetes status, hypertension status, education levels, zip code, and statin intake. All data were adjusted for the effects of weather by including temperature, barometric pressure, and season as covariates in the adjusted models, using dummies whenever appropriate. We performed stratified analyses by sex, age group and health status. We arbitrarily defined the healthy group as the ones who did not report, or have, any of the following conditions: diabetes, hypertension, smoking, asthma, allergy, chronic bronchitis, coronary artery disease (myocardial infarction, cardiac catheterization, coronary artery bypass surgery), heart failure, stroke, peripheral vascular disease, as well as any drug taken on a regular basis.

Descriptive statistical analysis used the Wilcoxon rank sum test (for medians). All data analyses were performed using Stata 12.0 software (StataCorp, College Station, TX, USA), and a two-sided significance level of 5 % was used.

## Competing interests

Vincent Mooser was a fulltime employee of GlaxoSmithkline.

## Authors' contributions

PV and GW performed the experiments and contributed to acquisition of data. DT, PMV, and MB analyzed the data and interpreted the data. The manuscript was written by DT, NA, and MB and revised critically by VM, MR, FP, and JW. All authors read, corrected and approved the final manuscript.

## Supplementary Material

Additional file 1: Table S1 Levels of inflammatory markers overall and by selected subgroups. **Table S2**. Change (and 95 % CI) in inflammatory markers associated with a 10 μg/m3 change in ambient 24 h average PM10 concentration. **Table S3**. Association of short-term exposure to 24 h average PM10 with inflammatory markers on the day of examination, and with 1-day to 6-day lags from adjusted robust regression models^§^. **Table S4**. Associations of short-term exposure to 24 h average PM10 with inflammatory markers, by selected strata. (PDF 208 kb) Click here for file
