# Supplementary material for: Effects of particulate matter on inflammatory markers in the general adult population
Source: Part Fibre Toxicol. 2012 Jul 6;9:24. doi: 10.1186/1743-8977-9-24 (PMC3464812; doi:10.1186/1743-8977-9-24)
Supplement: Additional file 1: Table S1 — Levels of inflammatory markers overall and by selected subgroups. Table S2. Change (and 95 % CI) in inflammatory markers associated with a 10 μg/m3 change in ambient 24 h average PM10 concentration. Table S3. Association of short-term exposure to 24 h average PM10 with inflammatory markers on the day of examination, and with 1-day to 6-day lags from adjusted robust regression models§. Table S4. Associations of short-term exposure to 24 h average PM10 with inflammatory markers, by selected strata. (PDF 208 kb) [file 1743-8977-9-24-S1.pdf]

Supplementary materials

Effects of particulate matter on inflammatory markers in the general adult population

Dai-Hua Tsai[1,2], Nadia Amyai[3], Pedro Marques-Vidal[1], Jia-Lin Wang[2], Michael Riediker[4], Vincent Mooser[5], Fred Paccaud[1], Gerard Waeber[3], Peter Vollenweider[3], Murielle Bochud[1§]

[1]Institute of Social and Preventive Medicine (IUMSP), Lausanne University Hospital (CHUV), Lausanne, Switzerland

[2]Department of Chemistry, National Central University, Taiwan

[3]Department of Medicine, Internal Medicine, CHUV, Lausanne, Switzerland

[4]Institute for Work and Health (IST), Lausanne, Switzerland

[5]Department of Genetics, GlaxoSmithKline, Philadelphia, PA, USA

Supplementary Material, Table 1. Levels of inflammatory markers overall and by selected subgroups

		hs-CRP (μg/mL)		IL-1β (pg/mL)		IL-6 (pg/mL)		TNF-α (pg/mL)	
	Subjects [n(%)]	N	Median (P25-P75)	N	Median (P25-P75)	N	Median (P25-P75)	N	Median (P25-P75)
**All subjects**	6183	6171	1.30(0.60-2.70)	3794	1.17(0.48-3.90)	5626	1.47(0.71-3.52)	6034	2.89(1.82-4.52)
**Sex**									
Female	3251(52.6)	3247	1.30(0.60-2.90)	2049	1.28(0.52-3.76)	2933	1.37(0.67-3.22)	3172	2.75(1.72-4.40)
Male	2932(47.4)	2924	1.20(0.60-2.60)	1745	1.08(0.45-3.96)	2693	1.59(0.77-3.79)	2862	3.05(1.90-4.65)
p-value			0.01		0.04		<0.01		<0.01
**Age (Years)**									
<55	3506(56.7)	3500	1.00(0.50-2.40)	2274	1.29(0.51-4.16)	3140	1.39(0.67-3.53)	3414	2.71(1.71-4.27)
>=55	2677(43.3)	2671	1.60(0.80-3.20)	1520	1.05(0.45-3.42)	2486	1.60(0.78-3.47)	2620	3.15(2.01-4.83)
p-value			<0.01		<0.01		<0.01		<0.01
**BMI**									
<25	2970(48.0)	2963	0.80(0.40-1.70)	1872	1.32(0.53-4.09)	2648	1.35(0.64-3.45)	2896	2.70(1.71-4.21)
>=25	3213(52.0)	3208	1.90(0.90-3.80)	1922	1.05(0.45-3.64)	2978	1.59(0.80-3.53)	3183	3.11(1.93-4.82)
p-value			<0.01		<0.01		<0.01		<0.01
**Smoking**									
No	4510(72.9)	4506	1.20(0.60-2.70)	2763	1.14(0.48-3.79)	4083	1.38(0.67-3.30)	4412	2.86(1.79-4.47)
Yes	1673(27.1)	1665	1.40(0.70-2.90)	1031	1.27(0.49-4.46)	1543	1.72(0.86-4.02)	1622	2.99(1.87-4.79)
p-value			<0.01		0.05		<0.01		0.01
**Diabetes**									
No	5764(93.2)	5764	1.20(0.60-2.60)	3588	1.19(0.49-3.91)	5237	1.45(0.70-3.46)	5635	2.86(1.79-4.48)
Yes	407(6.8)	407	2.10(1.00-4.50)	206	0.93(0.43-3.58)	388	1.93(0.92-3.86)	398	3.55(2.21-5.37)
p-value			<0.01		0.17		<0.01		<0.01
**Hypertension**									
No	3960(47.9)	3953	1.10(0.50-2.30)	2527	1.23(0.50-4.09)	3569	1.37(0.66-3.39)	3860	2.74(1.72-4.30)
Yes	2223(52.1)	2218	1.80(0.90-3.60)	1267	1.07(0.45-3.49)	2057	1.67(0.83-3.65)	2174	3.19(2.04-4.92)
p-value			<0.01		0.01		<0.01		<0.01
**Alcohol**									
No	4613(74.6)	4606	1.20(0.60-2.70)	2875	1.19(0.48-3.93)	4183	1.42(0.70-3.40)	4505	2.86(1.80-4.50)
Yes	1567(25.4)	1567	1.40(0.70-2.70)	918	1.14(0.47-3.78)	1441	1.59(0.79-3.94)	1527	2.99(1.86-4.61)
p-value			<0.01		0.36		<0.01		0.09

Note: by Wilcoxon rank sum test

Supplementary Material, Table 2. Change (and 95% CI) in inflammatory markers associated with a 10 μg/m^3 change in ambient 24h average PM_{10} concentration

	Crude effects	p-value	Adjusted effects [a]	p-value
**hs-CRP (μg/mL)**	0.0004 (-0.016, 0.016)	0.961	-0.002 (-0.017, 0.013)	0.820
**IL-1β (pg/mL)**	0.016 (-0.009, 0.040)	0.226	0.017 (-0.010, 0.044)	0.220
**IL-6 (pg/mL)**	0.026 (0.008, 0.043)	0.004	0.023 (0.005, 0.042)	0.014
**TNF-α (pg/mL)**	0.015 (0.004, 0.025)	0.005	0.024 (0.013, 0.035)	<0.001

[§] Covariates included in the model: sex, age, BMI, alcohol consumption, smoking status, education levels, zip code, statin, diabetes, hypertension, season, outdoor air temperature, and outdoor air pressure. Data are coefficients from robust regression models.

Supplementary Material, Table 3. Association of short-term exposure to 24h average PM10 with inflammatory markers on the day of examination, and with 1-day to 6-day lags from adjusted robust regression models§

	hs-CRP	p-value	IL-1β	p-value	IL-6	p-value	TNF-α	p-value
Lag 0 (Same day)	-0.002 (-0.017, 0.013)	0.820	0.017 (-0.010, 0.044)	0.220	0.023 (0.005, 0.042)	0.014	0.024 (0.013, 0.035)	<0.001
Lag 1	0.001 (-0.013, 0.016)	0.854	0.014 (-0.012, 0.041)	0.290	0.023 (0.005, 0.004)	0.013	0.022 (0.012, 0.033)	<0.001
Lag 2	0.002 (-0.013, 0.017)	0.798	0.020 (-0.007, 0.047)	0.146	0.026 (0.008, 0.044)	0.005	0.023 (0.013, 0.034)	<0.001
Lag 3	-0.001 (-0.015, 0.014)	0.944	0.017 (-0.010, 0.044)	0.207	0.029 (0.011, 0.047)	0.002	0.023 (0.012, 0.034)	<0.001
Lag 4	0.002 (-0.013, 0.017)	0.784	0.017 (-0.009, 0.043)	0.206	0.031 (0.012, 0.049)	0.001	0.022 (0.011, 0.033)	<0.001
Lag 5	0.003 (-0.011, 0.018)	0.662	0.017 (-0.010, 0.043)	0.221	0.029 (0.010, 0.047)	0.002	0.019 (0.008, 0.030)	0.001
Lag 6	0.002 (-0.012, 0.017)	0.753	0.013 (-0.013, 0.040)	0.325	0.031 (0.013, 0.050)	0.001	0.019 (0.008, 0.030)	0.001

§Adjusted for: sex, age, BMI, alcohol consumption, smoking status, education levels, zip code, statin, diabetes, hypertension, season, outdoor air temperature, and outdoor air pressure.

Supplementary Material, Table 4. Associations of short-term exposure to 24h average PM_{10} with inflammatory markers, by selected strata

	hs-CRP	p-value	IL-1ß	p-value	IL-6	p-value	TNF-α	p-value
**Female**	-0.008 (-0.028, 0.012)	0.439	0.010 (-0.026, 0.046)	0.582	0.012 (-0.013, 0.037)	0.354	0.023 (0.008, 0.038)	0.002
**Male**	0.009 (-0.013, 0.031)	0.416	0.028 (-0.013, 0.069)	0.183	0.038 (0.010, 0.066)	0.007	0.025 (0.009, 0.041)	0.002
**Interaction p-value§**		0.560		0.999		0.371		0.627
**Age<55**	-0.007 (-0.027, 0.013)	0.511	0.026 (-0.009, 0.061)	0.149	0.033 (0.008, 0.059)	0.011	0.027 (0.013, 0.042)	<0.001
**Age>=55**	0.007 (-0.015, 0.029)	0.527	0.004 (-0.039, 0.046)	0.866	0.013 (-0.014, 0.040)	0.360	0.020 (0.003, 0.037)	0.021
**Interaction p-value§**		0.404		0.315		0.079		0.624
**Healthy†**	0.025 (-0.009, 0.058)	0.151	0.048 (-0.017, 0.112)	0.150	0.062 (0.015, 0.108)	0.009	0.035 (0.009, 0.061)	0.009
**Non-healthy†**	-0.007 (-0.024, 0.009)	0.400	0.012 (-0.018, 0.041)	0.437	0.014 (-0.006, 0.034)	0.175	0.022 (0.010, 0.034)	<0.001
**Interaction p-value§**		0.620		0.331		0.391		0.778
**No statins**	-0.002 (-0.018, 0.014)	0.806	0.007 (-0.022, 0.036)	0.645	0.023 (0.003, 0.043)	0.027	0.024 (0.012, 0.036)	<0.001
**With statins**	0.006 (-0.036, 0.049)	0.773	0.083 (0.004, 0.161)	0.040	0.031 (-0.017, 0.081)	0.196	0.020 (-0.011, 0.051)	0.200
**Interaction p-value§**		0.843		0.130		0.699		0.809

Data are coefficients (95% CI) from robust regression models. Coefficients represent change in cytokine per 10 $\mu g/m^3$ increase in PM_{10}. § P-value for interaction between the strata variable and PM_{10} for their effects on the inflammatory marker.
† Sample sizes for healthy and non-healthy are 993 and 5190, respectively.
